# Challenges and perceptions of dental undergraduate students regarding active learning in clinical practice: A qualitative study

**DOI:** 10.1371/journal.pone.0328939

**Published:** 2025-09-16

**Authors:** Maria de Lara Araújo Rodrigues, Bruna Navarro Camargo da Silva, Marcella Alves dos Anjos, Eduarda Betiati Menegazzo, Álex Moreira Herval, Luciane Miranda Guerra, Jaqueline Vilela Bulgareli

**Affiliations:** 1 Department of Preventive and Social Dentistry, School of Dentistry, Federal University of Uberlândia, Uberlândia, MG, Brazil; 2 Department of Applied Psychology, Piracicaba Dental School, State University of Campinas, Piracicaba, SP, Brazil; Shahid Beheshti University of Medical Sciences School of Dentistry, IRAN, ISLAMIC REPUBLIC OF

## Abstract

**Objectives:**

This study aimed to investigate the challenges faced by dental students during clinical practice and to understand their perceptions of how active learning methodologies contribute to fostering critical and reflective participation in problem-solving throughout the teaching-learning process.

**Methods:**

This qualitative study applied the action research method in a descriptive and interpretive manner, implementing active learning methodologies in the adolescent clinic of the Federal University of Uberlândia (Brazil). This clinic is among the fundamental elements in the Public Health System. This research included 33 students in the final term of the dentistry course (10^th^ term), divided into three groups of 9–13 students each, and regularly enrolled in clinic practice. The data was collected in three 90-minute workshops with each group. This study used Minayo’s methodological framework and the arc method by Spanish educator Charles Maguerez to conduct the action research. This research comprised five steps: 1. Clinical experience; 2. Identification of challenges; 3. Theorization of challenges based on scientific evidence; 4. Development of action strategies; 5. Application of strategies in clinical practice. The theoretical framework was Paulo Freire’s problematization methodology. Data analysis followed the thematic content analysis technique.

**Results:**

After comprehensive data analysis, the main challenges concerned the work process, productivity, and infrastructure. The formulated strategies included the establishment of a procedures table, implementation of minimum age regulations for companions in the waiting area, adjustment of clinic hours, enhancement of auxiliary professional training, and maintenance of dental equipment.

**Conclusion:**

The groups’ experience with active methods qualified them to identify and address relevant practice challenges. It allowed study participants to lead their teaching-learning process and develop action strategies to transform the work process. Reflecting on implementing these strategies in clinical practice transformed the students’ reality and promoted more dynamic and meaningful learning. However, solving challenges, such as infrastructure, requires a continuous commitment of financial resources and institutional support.

## Introduction

The Brazilian National Curricular Guidelines for undergraduate dentistry courses, established in 2002 with the latest update in 2021, emphasize theory and practice integration. These guidelines advocate for training dental surgeons with comprehensive and humanistic health care, fostering critical and reflective thinking [1,2].

The pedagogical project of undergraduate dentistry courses must prioritize students as the primary learning agents and professors as facilitators and mediators [3,4]. Moreover, ethical training requirements include developing interpersonal skills, understanding health, illness, and care processes, and a critical perspective of reality [1,2,5].

Active learning methodologies are paramount, as they bridge the gap between theory and practice, promote a holistic individual perception, and expand the care concept [6,7]. Such methods support student autonomy [8] and comprehensive knowledge construction. The problem-solving methodology by Paulo Freire emphasizes learning contextualization, encourages the critical analysis of real-world problems, and promotes active student participation [8]. Investigating the problematization methodology in dental education is relevant, as it aligns with the need for critical thinking and practical skills in preparing competent professionals [6].

Integrating problem-solving into clinical education helps students develop the skills to handle complex cases effectively [9,10]. Using the problematization methodology as a teaching tool improves analytical thinking, autonomy, and student participation. This approach strengthens student relationships and empowers them to engage in their learning process actively [11–15].

Active dental education methodologies, such as problem-based learning (PBL), team-based learning (TBL), and case-based learning (CBL), enhance the teaching-learning process by promoting critical thinking, reasoning, and the application of clinical skills among dental students [10,13–15]. These approaches improve student engagement and help develop essential workplace skills, such as problem-solving [6].

Teaching and learning are central to clinical education and have been widely discussed, especially in medicine [16,17]. In dental education, clinical teaching and learning are essential for developing skills and improving the knowledge of dental students [18,19].

Brazilian dentistry courses aligned with the National Curricular Guidelines have increasingly incorporated active teaching-learning methodologies and small-group work to train health professionals [1,5]. However, limited research is available on dental students [20,21].

This study worked on the principle that clinical practice in primary health care can be improved and better understood through active learning strategies, which involve student engagement in educational processes. This assumption comes from Paulo Freire’s [8] pedagogical framework, particularly his problematization approach to understanding clinical care.

Therefore, the objective of this study was to investigate the challenges faced by dental students during clinical practice and to understand their perceptions of how active learning methodologies contribute to fostering critical and reflective participation in problem-solving throughout the teaching-learning process.

## Methods

The study design followed the guidelines of the Consolidated Criteria for Reporting Qualitative Research (COREQ). The COREQ is recommended for research reports collecting data through interviews or focus groups. It comprises 32 items divided into three domains: research team characterization and qualification, study design, and result analyses [22].

### Research team

The core research team comprised experienced qualitative methodologists. They were trained by a skilled researcher (J.V.B.). Three investigators, unknown to the participants, conducted the focus groups: a Master’s student (M.L.A.R.) as the moderator and two undergraduate students (B.N.C. and M.A.A.) as observers. They registered aspects of the participants’ non-verbal language and the sequence and content of speeches. The discussions were recorded using a mobile phone to facilitate subsequent transcription for content analysis.

The study underwent a pilot test to reduce potential errors in the method. The discussions from this test showed that participants might provide relevant information during group workshops. The training helped correct issues, such as the moderator’s posture, voice intonation, and the suitability of the semi-structured script application. The study sample included the group of nine participants, as the research script was unaltered.

### Study design and procedure

This is a qualitative study its methodological approach was based on Minayo [23,24]. This qualitative method emphasizes the dynamic interaction between researchers and participants. It aimed to understand the meaning given by the participants to the investigated phenomena.

Qualitative research does not aim for statistical generalization but rather focuses on interpretation and subjectivity [25,26]. It explores individual experiences and meanings within specific contexts [24].

This qualitative study fits into the framework of participatory action research, using focus groups as the primary data collection method. The stages of this research process are planning, action, observation, and reflection, involving the participants from the beginning [27,28].

Paulo Freire’s theoretical framework, as debated in various research papers, discusses the relevance of dialogue, critical thinking, and conscientization in education [29,30]. Paulo Freire was the theoretical foundation, with an interpretation centered on essential concepts of Freirean pedagogy, including liberating education, dialogue, and the conscientization process [3,8]. Integrating Freirean elements, such as dialogism and reflective thinking, into educational practices may foster transformative learning experiences that enhance students’ understanding and social changes [31].

### Participants and recruitment

The study targeted students in the primary health care clinic during the final term of the undergraduate dentistry course in Uberlândia, MG, Brazil. All study participants are paired and divided into four mentoring groups, each supervised by tutors.

This clinic assists adolescents aged 10–19 seeking dental care in primary health services. It offers preventive sealants, professional topical fluoride application (PTFA), extractions, and restorative, surgical, endodontic, and periodontal procedures. The selection of the adolescent clinic was made based on its applicability as a public health facility. The clinic is overseen by the university’s public health department, ensuring accessibility and continuity of care. The selection of this clinic is justified by its essential role in providing dental care in primary services to this population. The clinic emphasizes the importance of equitable access to oral health for the population.

The study used an intentional sampling technique, a methodological approach that often results in data saturation, where new observations become redundant [32]. The sample size was determined using the theoretical saturation method, an epistemological tool that marks the point at which additional data no longer expand the investigation or deepen understanding [32]. Once saturation was confirmed in the workshop phase, qualitative data analysis commenced immediately.

Additionally, thematic analysis of transcribed discussions validated the adequacy of the sample size with which to respond to the study’s objective, as well as ensuring alignment with the study’s theoretical framework [32]. This method is particularly recommended for focus groups [33], with the final sample size defined using the broad homogeneity criterion [34]. Research indicates that the average number of participants in each group is approximately 12 [33]. This study was conducted between June 22nd, 2023, and December 18th, 2023.

Study participants were personally invited to participate and given 15 days to respond and provide consent. Students were assigned alphanumeric codes (S01, S02, etc.) to ensure the confidentiality of their identities. Thirty-five individuals consented to participate in the study, with 33 attending all scheduled meetings. Two study participants who agreed to participate but were absent during the scheduled activity dynamics were excluded. The reason was a lack of interest in continuing the research. The inclusion criteria comprised study participants in the primary health care clinic who participated in all stages of the participatory action research process.

### Data collection

**Fig 1:**
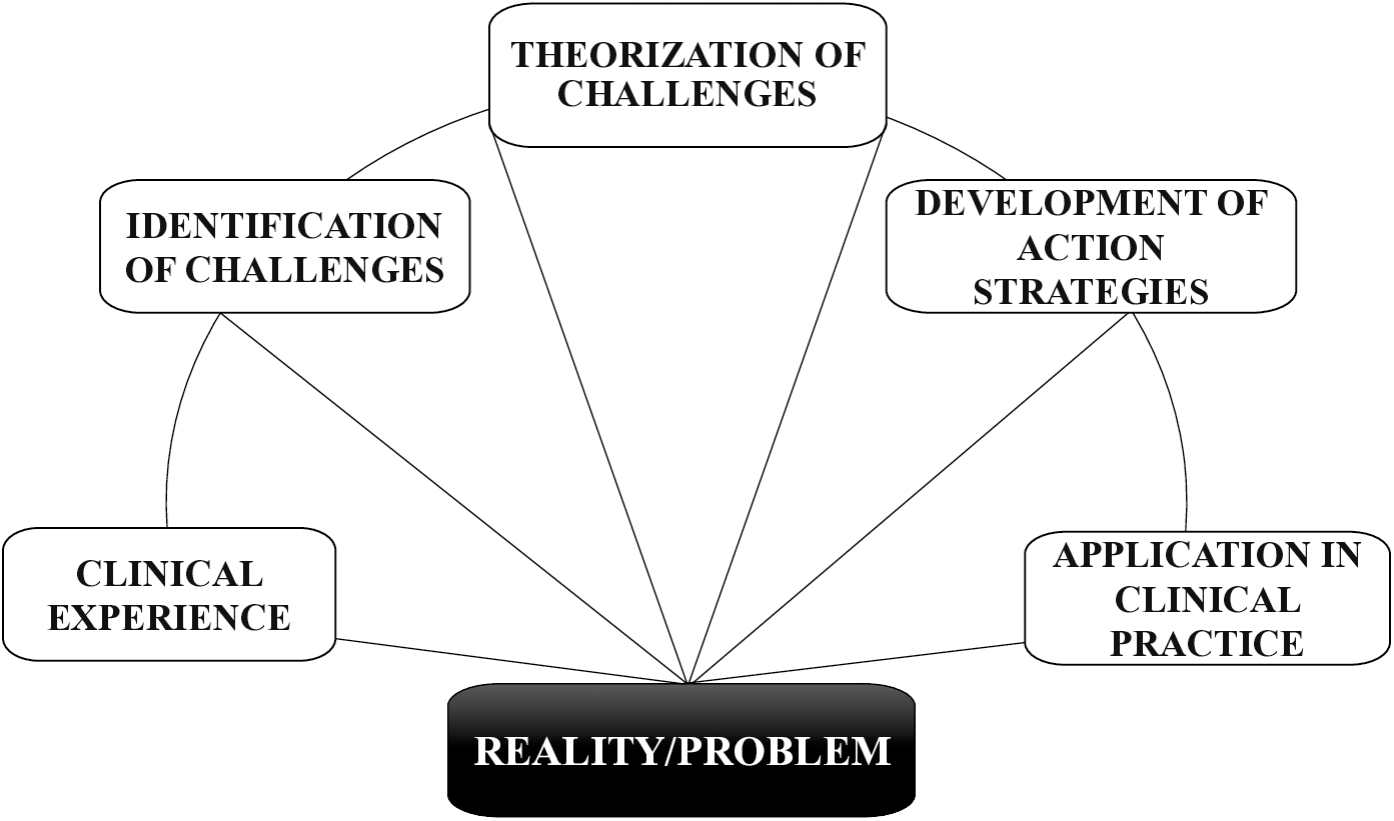
Illustrative adaptation of Charles Maguerez’s arc method.

The action research used Charles Maguerez’s arc method [35,36], which comprises five stages evolving from actual scenarios: 1. Clinical experience; 2. Identification of challenges; 3. Theorization of challenges; 4. Development of action strategies; 5. Application of strategies in clinical practice (Fig 1). This methodological trajectory facilitates data triangulation by integrating diverse sources and perspectives.

### Evaluation moments

**Fig 2:**
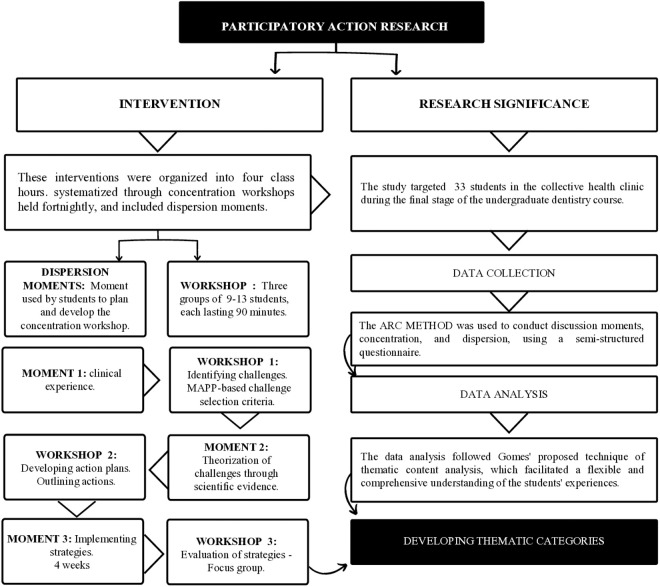
Illustrative action research path.

Interventions in the primary health care clinic were implemented to conduct the action research. These interventions were organized into four class hours, systematized through concentration workshops held fortnightly, and included dispersion moments. Three study groups sessions of 90 minutes each were conducted, concluding when achieving content saturation. Each study groups, consisting of 9–13 students, underwent three workshops with periods of face-to-face concentration. The final workshop for each group was a focus group to evaluate the action research process. Therefore, each of the three study groups conducted a focus group, completing a total of three focus groups in the study (Fig 2).

All workshops were recorded and performed in person at convenient times for the participants without disrupting the educational program schedule. This approach yielded valuable insights into clinical experience’s and practices at the dental school’s community health clinic.

### Incorporating active teaching strategies: An action research approach

#### Moment 1: Clinical experience.

During this stage, clinical experience comprised the active involvement of study participants in critically and reflectively evaluating the care provided at the primary health care clinic, and assessing the clinical reality. The focus was on identifying vital improvement areas, including interpersonal relationships, a lack of treatment adherence, insecurities, fears, anxieties, and other factors directly affecting clinical practice.

#### Workshop 1: Identifying challenges (concentration).

The selection criteria for the investigation/resolution challenges were determined using the modified Altadir method of popular planning (MAPP). This technique promotes a participatory and collaborative approach to community planning and development [37]. The MAPP stage of “problem selection” was used for problem identification. Participants within group settings identified the most urgent and significant issues requiring attention throughout the process to define future actions.

The aim was to investigate crucial issues in clinical practice using the following question: What are the main challenges in clinical practice?

The moderator developed a semi-structured form/questionnaire with guiding questions about the challenges dental study participants encountered during clinical training. Study participants identified three challenges and categorized them based on the following criteria: importance (high, medium, low), coping capacity (within, partial, outside), urgency level (very high, high, medium, low), and readiness to handle such challenges (yes or no).

The MAPP method considers a critical node the challenges classified as highly important, within the coping capacity, highly urgent, and ready to resolve. Solving these challenges might break the causal chain of the identified problem [38].

A series of group discussions helped the group analyze the primary challenges encountered in the clinic. These main difficulties led to the formulation of “challenges.” The objectives were the focal points, prompting study participants to conduct a literature search throughout the week. The findings were to be addressed in the subsequent stage of the study.

#### Moment 2: Theorization of challenges based on scientific evidence (dispersion).

This was an individual task to examine the challenges based on scientific evidence. The study participants had to review the literature critically, identify theories aligned with the challenges, and then apply these theories to clinical practice scenarios. The objective was to enhance student knowledge of the challenges and develop solutions based on the literature, stimulating critical thinking, creativity, and problem-solving.

#### Workshop 2: Developing action plans (concentration).

The second group conducted this phase, focusing on synthesizing the search for learning questions. Study participants engaged in critical and collective discussions about the theorization stage, facilitating the reflection on potential implementation strategies.

Study participants were organized in a discussion circle, and the moderator prompted them to provide feedback on their insights during challenge theorization. Study participants answered the following question: How did you feel when searching the literature for learning questions (problems)? Next, the moderator asked participants to summarize their research on learning questions to formulate action and intervention strategies collaboratively.

Under the moderator’s guidance, study participants developed a synthesis outlining intervention strategies for use in clinical practice. These action plans detailed the steps to achieve the goals, including required resources, timelines, and responsibilities. During the discussion, participants explored the practical implications of the strategies and exchanged ideas regarding adaptations.

#### Moment 3: Implementing strategies (dispersion).

Implementing strategies helped achieve the defined targets and establish the necessary background to ensure positive and sustainable results. Study participants had four weeks to implement the plans developed in the second clinical focus group.

#### Workshop 3: Evaluating strategies (focus group – concentration).

A third workshop was conducted as a focus group to evaluate action strategies and the entire action research process. Focus groups for data collection are an effective method to acquire detailed qualitative insights into a specific subject through participant engagement, allowing researchers to grasp participants’ viewpoints and opinions in a more nuanced and contextualized manner [39,40].

Assessing these strategies is essential to identify enhancement opportunities and gather participant responses. During this session, study participants were invited to provide general feedback on the progress of the proposed plans, organized in a discussion circle.

The study participants were prompted with the following questions:

- How did you feel about implementing the action strategies (solutions and proposals) in clinical practice?
- How did the proposed implementations contribute to transforming the clinical reality?
- How was your experience participating in the action research project?

### Data analysis

A researcher was responsible for transcribing the responses given by the academics into a spreadsheet that helped with the qualitative analysis procedure. Following the workshops, the discussions were transcribed verbatim in their original language to maintain the authenticity of the participants’ statements.

The thematic content analysis proposed by Gomes (2014) [41] helped interpret the qualitative data. Gomes delineates three main stages of content analysis: (a) thorough reading of the selected material; (b) exploration of the material; (c) development of an interpretative synthesis.

In accordance with the framework, an initial overview was conducted, facilitating the identification of pre-categories and the definition of theoretical concepts that underpin the analysis. Subsequently, the pre-categories were classified, and the central meanings within the analyzed excerpts were examined. The categories were then reorganized based on the identified themes, enabling the development of thematic writing that integrates the text’s meanings with the theoretical framework. Finally, the material was structured to establish connections between the themes, research objectives, and specific questions.

This approach assessed the experiences, emotions, and sensations of study participants during the action research cycle. It fosters a student-centered research environment, leading to more meaningful and impactful outcomes for all participants.

### Ethical approval and consent to participate

The study received approval from the Research Ethics Committee (CAAE: 68903023.80000.5152), and it followed the ethical norms and guidelines of Resolution nº 510 of April 7, 2016, of the Brazilian National Health Council. The study was also conducted according to Resolution nº 466 of December 12, 2012, which establishes the rules for studies involving human beings. These resolutions provide the main practices in human research, ensuring participants’ full rights. The participants received a Free and Informed Consent Form prepared by the research team to explain the research objectives, guaranteeing participant anonymity, minimizing research risks, and emphasizing the right to withdraw from participation at any time without repercussions. All participants provided written informed consent.

## Results

This research involved 33 dental students in the final year of the undergraduate dentistry course. The participants were divided into three groups of nine to 13 study participants each, regularly enrolled in clinic practice.

### Identification of challenges

Table 1 presents the main challenges classified according to the selection criteria of the modified MAPP. This phase identified problems based on the selection criteria for challenges. Relevant factors when prioritizing tasks are urgency level, coping capacity, and readiness to handle these challenges.

**Table 1:** Classification of challenges based on the selection criteria.

Selection criteria for challenges								
Challenge	Importance		Coping capacity		Urgency level		Readiness to handle challenges	
Distribution of dental materials.	High	22	Within	1	Very high	6	Yes	31
	Medium	11	Partial	21	High	15		
Time to fill out attendance forms (physical and electronic).	Low	0	Outside	11	Medium	12	No	2
					Low	0		
Distribution ofpatients and medical records	High	24	Within	0	Very high	10	Yes	32
Patients who miss appointments.	Medium	9	Partial	7	High	12		
Clinic hours compatible with school hours.	Low	0	Outside	26	Medium	10	No	1
					Low	1		
Sun exposure during clinical care.	High	23	Within	2	Very high	13	Yes	32
Equipment maintenance.	Medium	9	Partial	10	High	14		
No chairs for companions inside the clinic.	Low	1	Outside	21	Medium	6	No	1
Dental room cleanliness.					Low	0		

Although the group used the MAPP selection criteria, the main challenges were chosen by collective consensus. The challenges were the focal points, prompting study participants to search for knowledge in the scientific literature during the week, as they had to address their findings in the subsequent phase of the study.

### Action strategy development and implementation

After learning the challenges, the group developed intervention strategies for implementation in clinical practice. This included assigning responsibilities for each action and setting deadlines for development. These steps allow the evaluation of whether the plans were implemented or not. The main challenges were analyzed and categorized into three thematic areas: work process, productivity, and infrastructure (Table 2).

**Table 2:** Template of interventions according to thematic categories.

Thematic category	Challenge	Developed action strategy	Responsibility	Development deadline	Applied strategy	Action implementation (yes or no)
**Work process**	Distribution of patients and medical records.	Creation of a procedures table.	Professors, Master’s students, and secretaries.	4 weeks	Insertion of a daily list to check scheduled patients.	Yes
	Patients who miss appointments.	Development of an appointment scheduling tool.	Dental hospital and researchers.	24 months	Proposal submitted to the coordination to be explored in an additional study.	Partial
	Clinic hours compatible with school hours.	Request to change clinic hours.	Course coordination and dental hospital.	4 months	Change of clinic hours compatible with adolescent’s school hours.	Yes
	Access difficulties and errors in the dental hospital’s health information system.	Request to improve the dental hospital’s health information system.	Dental hospital.	4 months	Identification of main challenges for students and ways to improve the dental hospital system.	Partial
**Productivity**	Time to fill out attendance forms (physical and electronic).	Request to fill out forms exclusively electronically.	Dental hospital.	4 months	Implementation of electronic forms only.	Yes
	Distribution of dental materials.	Interview professionals at the dental materials distribution center.	Professors, Master’s students, and professionals from the dental materials distribution center.	12 months	Professionals require training. Stage developed in an additional study.	Partial
	Communication with the team to plan dental treatment.	Include clinical case discussions.	Course coordination, professors, and students.	4 months	Suggestions submitted to the Coordination for further studies.	No
**Infrastructure**	Sun exposure during clinical care.	Request to install protective film on the clinic’s windows, according to surveillance standards.	Course coordination and dental hospital.	4 months	Installation of protective film on the clinic’s windows.	Yes
	Equipment maintenance.	Request for dental equipment maintenance: X-ray machines, dental stools, and wastebasket. Purchase of more radiographic films.	Students and course coordination.	4 weeks	Performance of dental equipment maintenance. The Coordinator denied requests for more X-rays due to the high cost and because it is a Public Health System.	Yes
	No chairs for companions inside the clinic.	Except for the 1^st^ appointment and special cases, companions must wait in the waiting room.	Professors and students.	4 months	Application of the rule that companions must wait in the waiting room.	Yes
	Dental room cleanliness.	Request cleaning and disinfecting products for each dental room.	Students and course coordination.	4 months	Provision of cleaning and disinfecting supplies in each dental service room.	Yes

### Evaluating the action research process

Evaluating the action research process was significant in ensuring the effectiveness and relevance of the findings, as well as informing and enhancing future interventions. This strategy assessed the experiences, emotions, and sensations study participants encountered during the action research cycle, thereby promoting more meaningful and impactful outcomes.

Participants reflected on their experiences using the action research process by identifying the main challenges, finding solutions in the literature, and applying them to clinical practice. They critically evaluated the process and the impact of the actions taken during the study.

This analysis is presented in Table 3, which shows the thematic categories and their corresponding sub-themes, key findings, along with the most representative quotes that illustrate each theme.

**Table 3:** Themes and subthemes identified in analysis of the action research.

Thematic categories	Subthemes	Key Findings	Representative Quotes
**Dental care automatization**	Lack of critical thinking in undergraduate training	Students adopt a mechanistic approach due to insufficient reflection opportunities.	*“We become so automatic that we don’t even think about what we can improve...” (S11)*
	Depersonalization of work	Automatization reduces student agency, making them feel like mere parts of a system.	*“... things were so rushed for me and I didn’t notice what I could improve, you know?” (S23)*
**Problem-based learning as a teaching methodology in dental clinics**	Active problem-solving engagement	Students recognized the value of structured methodologies in addressing clinical challenges.	*“ There’s already a proposal to find a solution, you already have an appointment, a methodology to solve the problems.” (S21)*
	Student-centered approach	The methodology enabled students to identify and propose solutions independently.	*“ The problems came entirely from the students, right? There was no intervention from professors.” (S01)*
**Qualified listening as a therapeutic process in action research with undergraduates**	Emotional validation	Students felt heard and valued, enhancing trust and cooperation.	*“We feel heard, right? Because we’ve never had that...” (S14)*
	Identification of overlooked issues	Listening allowed participants to articulate concerns that would otherwise remain unnoticed.	*“ It’s an opportunity to talk about a few things that might be easy to resolve but aren’t because no one knows it’s a problem...” (S22)*

The subsequent section offers a comprehensive exposition of the thematic categories. This analysis emphasizes the students’ experiences and perceptions during the implementation of active methodologies in clinical practice.

### Thematic category 1: Dental care automatization

This category addresses the primary challenges study participants encounter when listing the issues during clinical practice. Some raised problems concerned a lack of preparation for critical thinking during the undergraduate process, creating a tendency towards a mechanistic approach to work processes.

Task automatization depersonalizes the work, making students feel like parts of a machine, unable to identify or suggest improvements. Furthermore, there is social pressure to be grateful for the circumstances.

This phenomenon indicates a significant absence of opportunities for reflection, particularly concerning automatization. The process may transform dental care into a mechanical routine, inhibiting constructive criticism and the pursuit of improvements.

“[...] *Assisting and finishing. It’s so intense that it sometimes blinds you to certain problems. I had a hard time thinking about what I was going to answer. I made an effort to do so, you know?*” (S8)

“[…] *Usually, we don’t have this in other clinics, a space to discuss the problems happening.*” (S10)

“*Until this activity, things were so rushed for me and I didn’t notice what I could improve, you know?*” (S23)

“*We become so automatic that we don’t even think about what we can improve, right? For example, when you asked what was there to improve, I found it very difficult, as many people get into our heads, saying ‘Oh, you have everything, you’re privileged, you have to be grateful and not complain’.*” (S11)

### Thematic category 2: Problem-based learning as a teaching methodology in dental clinics

This category demonstrated the potential integration of the problem-based learning-teaching methodology into dental clinics. Implementing action research significantly changed students’ perceptions of problem-solving abilities. This methodology is not merely a passive form of listening, it is regarded as an active commitment to the pursuit of solutions.

It indicates the capacity to engage students in problem-solving to promote a dynamic learning culture. Students are highly involved and understand their role in addressing challenges. Moreover, this category underscores the need for integrating this methodology into clinical teaching in higher education, as it engages study participants actively throughout the process.

“*There’s already a proposal to find a solution, you already have an appointment, a methodology to solve the problems.*” (S21)

“*What I found most interesting was the way each point was addressed, the methodology of listening to problems, applying them, and giving feedback.*” (S02)

“*It was a great opportunity to have a serious discussion about what was happening and raise problems, also providing solutions. The problems came entirely from the students, right? There was no intervention from professors.*” (S01)

“*I think it’s very important. It should actually be in all clinics [...] Some clinics need several changes.*” (S12)

### Thematic category 3: Qualified listening as a therapeutic process in action research with undergraduates

The final category aimed to learn student emotions throughout the action research process. The study participants felt welcomed and listened to during the action research process, helping to foster and create an environment of skilled listening. This promoted a sense of trust and cooperation among participants, nurturing a deeper level of engagement and commitment to research goals.

Study participants felt free to share their thoughts and ideas, knowing researchers valued and respected their contributions. This suggests that skilled listening facilitates articulating ideas and emotions and creates a sense of acknowledgment in individuals.

Furthermore, undergraduate study participants know the significance of addressing issues that might be overlooked. This indicates that effective listening allows the identification and resolution of problems that might remain unnoticed.

Despite their initial skepticism, undergraduate study participants appreciated the active teaching methods. Thus, such experiences may enhance student perceptions of alternative teaching strategies. Overall, the skilled listening environment contributed to a more meaningful and impactful research experience for all participants.

“*We feel heard, right? Because we’ve never had that. We’re graduating now and we’ve never had that. I thought it was very important and I even felt valued for participating in this moment and seeing things happen* [...]” (S14)

“*I wasn’t expecting much feedback so quickly. I think these are small things, but once you see them, they really make a difference* […]” (S19)

“*It’s an opportunity to talk about a few things that might be easy to resolve but aren’t because no one knows it’s a problem, no one is going through what we go through every day in the clinic.*” (S22)

“*I think it was a good thing because we felt very understood and listened to, and there were very quick solutions. It was one of the few times that I liked the active methodology. I don’t like it very much because our class hasn’t really picked up the active methodology. We’re from a past generation. I thought it was really cool that we did it. It was almost a therapy session.*” (S27)

## Discussion

This qualitative research encouraged using a problem-based methodology to handle challenges in clinical practice. The data was analyzed throughout the process to evaluate the proposed changes.

Education is a planned and participatory process of reflection and action, supporting groups to seek solutions and make decisions about problems affecting them, thereby meeting their needs and desires [42]. The participatory action research method facilitated a collaborative and reflexive approach. This methodology actively engages the participants in the research process [43]. The study participants in this study reported issues concerning the work process, productivity, and the university’s infrastructure.

The action research revealed that a problem-solving approach in clinical practice enhances active student engagement in the learning process, promoting debate, inquiry, and critical reflection. Saul (2016) [44] discusses studies using Freirean theoretical foundations in continuing education. Ongoing training requires a critical reflection on practice to analyze and understand its various determinations and relationships [44].

Participants faced difficulties in identifying critical issues in clinical practice, verified in the thematic category of “Dental care automatization.” This may be due to participants’ familiarity with the traditional banking methodology model used by the institution. Benitez (2023) [2] argues that conventional pedagogy does not meet the expectations of the teaching-learning process. There is a continuous debate in Brazil about the need to change traditional health education models to more active, innovative, and student-centered approaches [20,28,29].

Action research is a methodological tool used in educational practice and problem-posing education. Hence, investigating the thought process is crucial [8]. The traditional method may be perceived as limiting in promoting critical and participatory thinking among study participants. The critical thinking concept, as advocated by the educator Paulo Freire, represents a unique, singular, and continuous process in pedagogical practice [31].

The research findings align with the existing literature and indicate the need for transitioning towards interactive and engaging teaching methodologies. The research-teaching nexus is constantly adapting to the ever-changing landscape of the dynamic between educators and students [15,16]. This shift may yield significant benefits for student learning. According to the Brazilian National Curricular Guidelines, health courses such as dentistry and medicine advocate active methods and small group work to train health professionals, enriching the teaching-learning process in undergraduate courses [1,45,46].

Reflecting on the interaction interfaces between different players, such as students and healthcare teams (professors, secretaries, and coordinators), the clinical practice experience exposed a gap in the necessary interconnection regarding student training. This condition is associated with universities’ teaching models of prioritizing traditional teaching methods and producing clinical procedures over the learning process. In this context, Botazzo [47] highlights the theory of buccality. A broader understanding of oral health is required beyond the exclusive technical language of the dental field [47,48]. The lack of preparation for critical thinking during undergraduate education may promote a mechanistic approach to work processes.

The thematic category of “Problem-based learning as a teaching methodology in dental clinics” indicated a notable transformation in student perceptions of problem-solving abilities. This shift has been attributed to problematization methodologies in the educational context. This approach has also facilitated the potential development of essential skills for student training, such as critical thinking and collaboration. Although institutions acknowledge the National Curricular Guidelines with pedagogical trends that promote learning centered on critical, reflective, and transformative pedagogy, these principles are not yet visibly applied in the pedagogical projects of dentistry courses [2].

Most proposed actions were implemented through active student participation throughout the process. They established criteria and identified challenges to develop, implement, and evaluate strategies. This study highlights the relevance of incorporating this methodology into the curriculum of clinical activities in higher education institutions. Active teaching-learning methods represent pedagogical approaches based on problem-solving pedagogy, which places students in the center of the teaching-learning process and involves them in discovering, investigating, or solving problems [15,49].

Berbel (1998) [50] stated that the problematization methodology does not guarantee predictable results. The study participants encountered barriers when attempting to implement some strategies. Specifically, they faced challenges with strategies outside their governance scope, such as creating a scheduling tool and distributing dental supplies. Consequently, these strategies were not implemented during the process.

The action research process fostered a closer relationship between study participants and educators through the integration network facilitated by feedback provision. The feedback mechanisms inherent to clinical practices of undergraduate health courses are vital to the learning process [51,52].

The thematic category of “Qualified listening as a therapeutic process in action research with undergraduates” demonstrated that study participants acknowledge the significance of the methodology. Active listening offers an opportunity to situate students at the center of the teaching-learning process. This allows educators to encourage collaborative strategies and provide a welcoming environment, contrasting with the current educational model of institutions. Active listening provides students with the opportunity to verbalize their anxieties and receive emotional support [53,54]. Acknowledging health students is an essential strategy to promote a conducive and productive learning environment, contributing to academic success and the professional development of future healthcare practitioners [9,49].

Qualified training stimulated by critical thinking development in future healthcare professionals contributes to the guidelines of the National Oral Health Policy [55]. Law 14.572/2023 incorporated this policy into the Brazilian Unified Health System (SUS) to facilitate the universalization of oral health care access, guaranteeing the benefits of quality dental care to the population [55].

Implementing problem-based methodologies has promoted a more adequately prepared student cohort to face the clinical challenges of primary health care with a generalized approach. Hence, students are equipped to provide excellent care to the population [56].

Developing and implementing the strategies outlined by the study participants in this study required substantial financial and institutional support. This aspect aims to integrate essential oral health care into primary health services. Such integration seeks to ensure financial protection and adequate supply provision, expanding oral healthcare access in a safe, effective, and affordable manner [57].

Student engagement with the problem-based learning methodology may be associated with their developmental progression, especially in the final term of their course. Those in advanced stages of the academic journey tend to be more actively involved in the teaching-learning process, promoting more integration [20].

This research evidenced that implementing active teaching-learning methods fosters a critical and problem-solving mindset in students. These active methodologies create an environment where students construct their knowledge, thereby prompting reflection on the practical scenarios they encounter.

### Strengths, limitations and future research

One of the key strengths of study lies in its participatory approach, which empowered students to take an active role in identifying and addressing real-world clinical challenges. Another strength is that students felt heard and supported in their needs. However, this study has certain limitations. This study’s qualitative nature limits result generalizability. Additionally, action research requires significant time, posing challenges to implementing suggested strategies. Future research should further explore problematizing methodologies in dentistry and other clinical fields, enhancing theory-practice integration in university education.

## Conclusions

The present study significantly contributed to higher education in the health field. Active methodologies in clinical practice made study participants more engaged in addressing clinical challenges. Students led their learning process by developing and implementing strategies to improve their clinical practice. Reflecting on these strategies transformed their reality and promoted a dynamic and meaningful learning process. However, it highlighted the need for constant investments in institutional infrastructure and support to guarantee the implementation of this methodology and address persistent challenges.

## References

[1] Conselho Nacional de Educação/Câmara de Educação Superior. Resolução CNE/CES nº 3, de 21 de junho de 2021 - Institui as Diretrizes Curriculares Nacionais do curso de graduação em Odontologia e dá outras providências, publicado no D.O.U. de 2021 Jun 21 [cited 2023 Jan 23]; Seção 1. 59 p. Available from: http://portal.mec.gov.br/index.php?option=com_docman&view=download&alias=191741-rces003-21&category_slug=junho-2021-pdf&Itemid=30192

[2] Benitez JFD, Mesquita ATM, Benitez MO, Miranda JL de. A influência das Diretrizes Curriculares Nacionais nos cursos de graduação em odontologia. Acervo Saúde. 2023;23(5):e12448. doi: 10.25248/reas.e12448.2023

[3] Lage RH, Almeida SKTT de, Vasconcelos GAN, Assaf AV, Robles FRP. Ensino e aprendizagem em Odontologia: análise de sujeitos e práticas. Rev Bras Educ Med. 2017;41(1):22–9. doi: 10.1590/1981-52712015v41n1rb20150155

[4] Noro L. Como estruturar um currículo integrado num curso de odontologia? Rev Ciênc Plur. 2019;5(1):1–17. doi: 10.21680/2446-7286.2019v5n1id17942

[5] Moimaz AS, Custodio BM, Saliba TA, Garbin AJI, Garbin CAS. Diretrizes curriculares nacionais para o curso de odontologia: uma análise sob a ótica da estrutura textual. Rev Ens Educ Cienc Hum. 2021;21(4):508–13. doi: 10.17921/2447-8733.2020v21n4p508-513

[6] Mitre SM, Siqueira-Batista R, Girardi-de-Mendonça JM, de Morais-Pinto NM, Meirelles C de AB, Pinto-Porto C, et al. Active teaching-learning methodologies in health education: current debates. Cien Saude Colet. 2008;13 Suppl 2:2133–44. doi: 10.1590/s1413-81232008000900018 19039397

[7] Carvalho RNG de, Demberg RR, Ferraz RM, Santos IP dos, Pereira E da S, Silva JR, et al. Metodologias ativas para a aprendizagem na Instituição de Ensino Superior. RSD. 2022;11(12):e293111234614. doi: 10.33448/rsd-v11i12.34614

[8] Freire P. Pedagogia da autonomia: saberes necessários à prática educativa. 74a ed. Editora Paz e Terra; 2019.

[9] Burgess A, van Diggele C, Roberts C, Mellis C. Key tips for teaching in the clinical setting. BMC Med Educ. 2020;20(Suppl 2):463. doi: 10.1186/s12909-020-02283-2 33272257 PMC7712575

[10] Perez A, Howey M, Green JL, Nóbrega MTC, Kebbe M, Amin M, et al. Multiple cases in case-based learning: a qualitative description study. Eur J Dent Educ. 2023;27(4):1067–76. doi: 10.1111/eje.12900 36776122

[11] Rêgo HMC, Rodrigues JR. Methodology of problematization with the maguerez’s arch: an alternative method for teaching, research and study in dentistry. BDS. 2015;18(1):34–43. doi: 10.14295/bds.2015.v18i1.1047

[12] Ali K, Daud A, Ba Hattab R, Philip N, Matoug-Elwerfelli M, Anweigi L, et al. Development of self-regulation amongst dental students in problem-based learning curricula: a qualitative study. Eur J Dent Educ. 2023;27(2):388–95. doi: 10.1111/eje.12820 35579047

[13] Sekhon TS, Sekhon S, Gambhir RS. Students’ preferences regarding teaching methodology in dental education - a cross-sectional study. Przegl Epidemiol. 2022;76(2):210–5. doi: 10.32394/pe.76.21 36218166

[14] Miranda KC, Abreu MFF de, Bardini VS dos S, Tango RN, Vasconcellos LMR, Salgado MAC, et al. New teaching strategies in dentistry. Rev ABENO. 2022;22(2):1526. doi: 10.30979/revabeno.v22i2.1526

[15] Paiva MRF, Parente JRF, Brandão IR, Queiroz AHB. Metodologias ativas de ensino aprendizagem: revisão integrativa. SANARE. 2016;15(2):145–53. Available from: https://sanare.emnuvens.com.br/sanare/article/view/1049

[16] Howell K. Enhancing research and scholarly experiences based on students’ awareness and perception of the research-teaching nexus: a student-centred approach. PLoS One. 2021;16(9):e0257799. doi: 10.1371/journal.pone.0257799 34570801 PMC8475989

[17] Khani H, Ahmady S, Sabet B, Namaki A, Zandi S, Niakan S. Teaching-learning in clinical education based on epistemological orientations: a multi-method study. PLoS One. 2023;18(11):e0289150. doi: 10.1371/journal.pone.0289150 38032997 PMC10688630

[18] Ross J, Holder A. Dental undergraduate students’ perceptions about placements in primary dental care during the undergraduate dental curriculum: a qualitative evidence synthesis. Br Dent J. 2022;233(2):141–7. doi: 10.1038/s41415-022-4457-6 35869216

[19] Javed MQ, Nawabi S, Srivastava S, Kolarkodi SH, Khan AM, Awinashe MV. Undergraduate students’ and interns’ perception towards learning environment at dental clinics, Qassim University, Saudi Arabia. J Pharm Bioallied Sci. 2023;15(Suppl 1):S419–25. doi: 10.4103/jpbs.jpbs_562_22 37654370 PMC10466525

[20] Gontijo LPT, Herval ÁM, Carcereri DL, Freitas SFT de. Aceitabilidade das metodologias ativas de ensino-aprendizagem entre discentes de odontologia. RIAEE. 2020;15(4):2023–48. doi: 10.21723/riaee.v15i4.13693

[21] Dargue A, Richards C, Fowler E. An exploration of the impact of working in pairs on the dental clinical learning environment: Students’ views. Eur J Dent Educ. 2023;27(1):87–100. doi: 10.1111/eje.12780 35100467 PMC10078664

[22] Tong A, Sainsbury P, Craig J. Consolidated criteria for reporting qualitative research (COREQ): a 32-item checklist for interviews and focus groups. Int J Qual Health Care. 2007;19(6):349–57. doi: 10.1093/intqhc/mzm042 17872937

[23] Minayo MC (org), Deslandes SF, Gomes R. Pesquisa social: teoria, método e criatividade. 34a ed. Ed Vozes; 2014.

[24] Minayo MC de S. Qualitative analysis: theory, steps and reliability. Cien Saude Colet. 2012;17(3):621–6. doi: 10.1590/s1413-81232012000300007 22450402

[25] Mattos PLCL de. “Os resultados desta pesquisa (qualitativa) não podem ser generalizados”: pondo os pingos nos is de tal ressalva. Cad EBAPEBR. 2011;9(spe1):450–68. doi: 10.1590/s1679-39512011000600002

[26] Valle PRD, Ferreira JDL. Análise de conteúdo na perspectiva de bardin: contribuições e limitações para a pesquisa qualitativa em educação. Educ Rev. 2025;41:e49377. doi: 10.1590/0102-469849377

[27] Corrêa GCG, Campos ICP de, Almagro RC. Pesquisa-ação: uma abordagem prática de pesquisa qualitativa. Ens Ped. 2018;2(1):62–72. doi: 10.14244/enp.v2i1.60

[28] Souza AMC, Silva ITC, Guimarães AGDP. Pesquisa-ação: um olhar para a atenção primária em saúde. Rev Sau Aer. 2021;4(3):17–23.

[29] Araújo BBM de, Machado ADCC, Rossi CS, Pacheco ST de A, Rodrigues BMRD. Referencial teórico-metodológico de Paulo Freire: contribuições no campo da enfermagem [Paulo Freire’s theoretical and methodological framework: contributions in the field of nursing] [Referencial teórico-metodológico de Paulo Freire: contribuciones en el campo de la enfermería]. Rev Enferm UERJ. 2018;26:e27310. doi: 10.12957/reuerj.2018.27310

[30] Roberts P. Paulo freire on democratic education. In: Culp J, Drerup J, Yacek D, editors. The cambridge handbook of democratic education. Cambridge: Cambridge University Press; 2023. p. 90–107.

[31] Martins MA das NS, Diniz Abreu TC, Moura LNS de. Práxis freireana: diálogo, pesquisa-ação e escola democrática. OlharProfr. 2020;23:1–15. doi: 10.5212/olharprofr.v.23.2020.16401.209209227954.0715

[32] Fontanella BJB, Ricas J, Turato ER. Saturation sampling in qualitative health research: theoretical contributions. Cad Saude Publica. 2008;24(1):17–27. doi: 10.1590/s0102-311x2008000100003 18209831

[33] Souza LK de. Recomendações para a Realização de Grupos Focais na Pesquisa Qualitativa. PSIUNISC. 2020;4(1):52–66. doi: 10.17058/psiunisc.v4i1.13500

[34] Turato ER. Tratado da metodologia da pesquisa clínico-qualitativa: construção teórico-epistemológica, discussão comparada e aplicação nas áreas da saúde e humanas. 6th ed. Petrópolis (RJ): Vozes; 2013.

[35] Berbel NAN. As metodologias ativas e a promoção da autonomia de estudantes. Semin Cienc Soc Hum. 2011;32(1):25–40. doi: 10.5433/1679-0383.2011v32n1p25

[36] Prado ML do, Velho MB, Espíndola DS, Sobrinho SH, Backes VMS. Arco de Charles Maguerez: refletindo estratégias de metodologia ativa na formação de profissionais de saúde. Esc Anna Nery. 2012;16(1):172–7. doi: 10.1590/s1414-81452012000100023

[37] Baldissera VDA, Góes HLF. The altadir method of popular planning as a management teaching instrument in nursing. Invest Educ Enferm. 2012;30(2):253–9.

[38] Anjos EB dos, Oliveira ALA, Costa ALB, Cunha DG, Franco JM, Souza WF de. Método Altadir de Planificação Popular – mapp; atenção à gestante em uma unidade de saúde da família: um relato de experiência. In: Ciências da saúde: desafios, perspectivas e possibilidades - Vol 2. Editora Científica Digital; 2021. p. 210–8. doi: 10.37885/210605159

[39] Oliveira SFD, Machado FCDA. Percepção dos profissionais da estratégia saúde da família sobre processos educativos em saúde. Rev Ciênc Plur. 2020;6(1):56–70.

[40] Barbosa A de S, Teixeira BRF, Oliveira AM, Pessoa TRRF, Vaz EMC, Forte FDS. Interprofissionalidade, formação e trabalho colaborativo no contexto da saúde da família: pesquisa-ação. Saúde Debate. 2022;46(spe5):67–79. doi: 10.1590/0103-11042022e506

[41] Gomes R. Análise e interpretação de dados de pesquisa qualitativa. In: Minayo MC (org), Deslandes SF, Gomes R, editors. Pesquisa social: teoria, método e criatividade. 34a ed. Ed Vozes; 2014.

[42] Toledo RF de, Jacobi PR. Pesquisa-ação e educação: compartilhando princípios na construção de conhecimentos e no fortalecimento comunitário para o enfrentamento de problemas. Educ Soc. 2013;34(122):155–73. doi: 10.1590/s0101-73302013000100009

[43] Silva AAF, Oliveira GS, Ataídes FB. Pesquisa-ação: princípios e fundamentos. Rev Prisma. 2021;2(1):2–15.

[44] Saul AM, Saul A. Contribuições de Paulo Freire para a formação de educadores: fundamentos e práticas de um paradigma contra hegemônico. Educ Rev. 2016;61:19–35. doi: 10.1590/0104-4060.46865

[45] Brasil. Ministério da Educação. Conselho Nacional de Educação. Câmara de Educação Superior. Resolução Nº. 3 de 20 de junho de 2014. Institui diretrizes curriculares nacionais do curso de graduação em Medicina e dá outras providências. Brasília: Diário Oficial da União; 2014 Jun 23; Seção 1. p. 8–11.

[46] Ferreira MJM, Ribeiro KG, Almeida MM de, Sousa M do S de, Ribeiro MTAM, Machado MMT, et al. New National Curricular Guidelines of medical courses: opportunities to resignify education. Interface (Botucatu). 2019;23(suppl 1). doi: 10.1590/interface.170920

[47] Botazzo C. Diálogos sobre a boca. São Paulo: Hucitec; 2013.

[48] Fonsêca GS, Junqueira SR, Botazzo C, Carvalho YM de, Araujo ME de. A clínica do corpo sem boca. Saude soc. 2016;25(4):1039–49. doi: 10.1590/s0104-12902016163946

[49] Bacich L, Moran J, organizadores. Metodologias ativas para uma educação inovadora: uma abordagem teórico-prática [recurso eletrônico]. Porto Alegre: Penso; 2018.

[50] Berbel NAN. A problematização e a aprendizagem baseada em problemas: diferentes termos ou diferentes caminhos? Interface (Botucatu). 1998;2(2):139–54. doi: 10.1590/s1414-32831998000100008

[51] Hew KF, Lo CK. Flipped classroom improves student learning in health professions education: a meta-analysis. BMC Med Educ. 2018;18(1):38. doi: 10.1186/s12909-018-1144-z 29544495 PMC5855972

[52] Ghassan A, Shukr I, Sadiq N, Ahsan R. Current trends in dental education. PAFMJ [Internet]. 2021 [cited 2024 Mar 16];71(3):1107–13. doi: 10.51253/pafmj.v71i3.6318

[53] Siqueira MAM, Gonçalves JP, Mendonça VS, Kobayasi R, Arantes-Costa FM, Tempski PZ, et al. Relationship between metacognitive awareness and motivation to learn in medical students. BMC Med Educ. 2020;20(1):393. doi: 10.1186/s12909-020-02318-8 33126882 PMC7602298

[54] Penha JRL, Oliveira CC de, Teles Bezerra EA, Neto JC. A escuta qualificada como terapêutica no atendimento entre universitários: um relato de experiência. Biomotriz. 2020;14(3):127–36. doi: 10.33053/biomotriz.v14i3.38

[55] Brasil. Lei Nº 14.572, de 8 de maio de 2023. Institui a Política Nacional de Saúde Bucal no âmbito do Sistema Único de Saúde (SUS) e altera a Lei nº 8.080, de 19 de setembro de 1990, para incluir a saúde bucal no campo de atuação do SUS. Diário Oficial da União; 2023 maio 9; Seção 1:1.

[56] Peduzzi M, Norman IJ, Germani ACCG, da Silva JAM, de Souza GC. Interprofessional education: training for healthcare professionals for teamwork focusing on users. Rev Esc Enferm USP. 2013;47(4):977–83. doi: 10.1590/S0080-623420130000400029 24310699

[57] World Health Organization. Global strategy and action plan on oral health 2023–2030. Geneva: World Health Organization; 2024. Licence: CC BY-NC-SA 3.0 IGO.

